# Peer-assisted telemedicine hepatitis-C treatment for people who use drugs in rural communities: a mixed methods study

**DOI:** 10.1186/s13722-025-00541-6

**Published:** 2025-02-08

**Authors:** Kim Hoffman, Gillian Leichtling, Sarah Shin, Andrew Seaman, Tonhi Gailey, Hunter C. Spencer, P. Todd Korthuis

**Affiliations:** 1 Department of Medicine, Division of General Internal Medicine & Geriatrics, Section of Addiction Medicine, Oregon Health & Science University, Portland, OR USA; 2 Comagine Health, Portland, OR, USA; 3 Oregon Health & Science University, Portland, OR USA

## Abstract

**Background:**

The increase in opioid use disorder among young, nonurban people has fueled sharp rises in hepatitis C virus (HCV) infections. Innovative treatment models are needed that circumvent healthcare system barriers for people who use drugs (PWUD), particularly in rural areas. The Oregon HOPE TeleHCV study randomized PWUD living with HCV in rural Oregon to peer-facilitated and streamlined telemedicine HCV treatment (Peer TeleHCV) versus enhanced usual care (EUC) and assessed sustained virologic response at 12 weeks post treatment (SVR12). Peer Support Specialists (peers) conducted HCV screening in the community, facilitated pretreatment evaluation and linkage to telemedicine HCV treatment clinicians, and supported Peer TeleHCV study participants in HCV medication adherence. A qualitative investigation queried telemedicine clinicians and peers about their experiences with the implementation of the model and key drivers of implementation effectiveness.

**Methods:**

Two remote audio/video recorded focus groups were conducted, one with the study’s clinicians and one with the peers. Participants were asked their views of key elements for successful implementation and outcomes of the Peer TeleHCV model. Group interviews lasted one hour. Recordings were professionally transcribed for thematic analysis with a mixed deductive and inductive framework, using Atlas.ti. Patients were surveyed about their interactions and satisfaction with peers.

**Results:**

Quantitative data (n = 78) indicated patients had high levels of satisfaction with and support from the peers. Three themes were identified from the qualitative data (n = 12) including. (1) Key peer-level elements such as providing support during potentially difficult lab draws, creating a peer-facilitated “bubble of trust” between patients and clinicians, enabling technology access, conducting outreach to maintain contact and support treatment retention, and facilitating stabilizing wrap-around services (e.g., housing vouchers) (2) Key clinician-level factors such as capacity for unscheduled peer-facilitated appointments, having dedicated time for case consults with peers, and clinicians trained in working with PWUD and skilled in identifying related clinical concerns (3) Key systems-level elements such as standing lab orders, challenges related to specialty pharmacies and Medicaid managed care organizations, and streamlined communication strategies between peers and clinicians.

**Conclusion:**

All participants reported that the Peer TeleHCV model built trust and eased barriers for PWUD initiating and remaining in HCV treatment. This low-barrier model makes space for PWUD to receive HCV treatment, regardless of drug use. Implementing support from peer specialists, telemedicine technology, and streamlined testing and treatment strategies may connect more rural PWUD living with HCV with the cure.

## Introduction

The increase in opioid use disorder among young, non-urban people has fueled sharp rises in hepatitis C virus (HCV) infections [1–3]. Rural communities have limited treatment and harm reduction services and face access barriers to HCV treatment, including transportation and housing instability [4,5]. Flexible, non-stigmatizing treatment options designed for engaging people who use drugs (PWUD) in HCV treatment are needed [6].

Highly effective treatments for HCV have been available for more than a decade [7]. PWUD who receive HCV treatment have lower HCV transmission rates, decreased high-risk substance use practices, and increased substance use treatment completion rates [8–10]. However, PWUD often find it difficult to access HCV treatment due to drug use stigma from clinicians, long wait times to see an HCV specialist, few case management or peer support programs to facilitate access to care, and payment and reimbursement challenges [11–13]. Individuals in rural areas have the additional barrier of long travel times to already limited clinicians. [14]

Despite advancements in telemedicine as a result of the COVID-19 pandemic [15], innovative treatment models are needed that circumvent healthcare system barriers for PWUD, particularly in rural areas. Few studies examine how to engage PWUD who are not in either primary care or substance use disorder (SUD) treatment. Peer support specialists (peers) present an opportunity to bridge this gap as they leverage lived experience with substance use and engage non-treatment-seeking individuals by fostering trust and facilitating care coordination [16–20]. Most states, including Oregon, have limited Medicaid reimbursement options for peer services outside of SUD treatment episodes, and the work is often funded by grants [21]. The Oregon HOPE TeleHCV study employs peers who engage PWUD to offer HCV screening and linkage to HCV treatment and to provide support for factors (e.g., access to a cellphone, housing support) that can impede treatment uptake in PWUD [22]. The study randomized PWUD living with HCV in rural Oregon to peer-facilitated and streamlined telemedicine HCV treatment (Peer TeleHCV) versus enhanced usual care (EUC) [23]. Study peers assisted uninsured Peer TeleHCV study participants with Medicaid enrollment (insurance eligibility was a requirement for study participation), facilitated connection to HCV testing in the community, facilitated pretreatment evaluation and linkage to telemedicine HCV treatment clinicians, and supported Peer TeleHCV study participants in HCV medication adherence and completion of post-treatment labs to confirm cure (sustained viral response 12 weeks after treatment; SVR12). The telemedicine clinician team included two physicians, a nurse practitioner, and a clinical pharmacist who provided availability for “walk-in” virtual visits Monday through Friday afternoons, and who were experienced in serving people who use drugs and vulnerable populations. (Clinical pharmacists practicing in health care settings in Oregon are able to provide telemedicine as part the patient’s care team.) The randomized controlled trial found that Peer TeleHCV participants were 7 times more likely to be treated and 4 times more likely to be cured than EUC participants [24]. The goal of the current study was to understand peer, clinician, and Peer TeleHCV study participants views of Peer TeleHCV treatment model implementation.

## Methods

A peer support satisfaction survey and qualitative focus groups were performed.

*Qualitative Methods.* Two remote audio recorded focus groups were conducted from June to July 2022 with all staff who had implemented the intervention, one with clinicians (n = 5) and one with peers (n = 7). Focus groups lasted approximately 60 min following provision of verbal consent. Focus group discussions were facilitated by an experienced study staff (KH) [25–29] Participants completed a preliminary survey to identify key elements of the model to inform the interview guide and discussion. A semi-structured interview guide facilitated discussion of participants’ (1) views of key elements for successful implementation and outcomes, (2) barriers and facilitators they encountered, and (3) suggestions for improving the model. Participants did not receive an incentive. Recordings were professionally transcribed and analyzed using Thematic Analysis [30] with a semantic, inductive approach to identify themes. A thematic coding framework was developed by three members of the study team (KH, GL, SS). ATLAS.ti Web (v4.6.0–2022-12-06) qualitative software facilitated coding, organization, and retrieval of text for analysis. Transcripts were jointly coded (KH and SS) and a third study team member (GL) joined as the analysts reviewed the coding and further refined the application of the thematic codes for analysis. The study team met weekly to construct and review themes, which were then discussed during project team meetings. The study was reviewed and approved by the Oregon Health and Sciences University Institutional Review Board (IRB# 20,911). All participants consented to participate and the research was conducted in accordance with the Declaration of Helsinki.

### Peer support satisfaction assessment survey methods

We conducted a self-reported satisfaction survey with Peer TeleHCV study participants to better understand their perspective of peer support provided by the model. Participants completed a survey administered by telephone at 12 weeks post-treatment, the timepoint at which phlebotomy is conducted to assess the trial’s outcome of sustained virologic response (SVR12). The survey included seven items examining experiences and satisfaction with services provided by the study peers. Survey items were adapted for the study from the Patient Experiences Questionnaire for Interdisciplinary Treatment for Substance Dependence (PEQ-ITSD) [31], health-related Information Exchange Questionnaire (IEQ) [32], and South African Addiction Treatment Services Assessment (SAATSA) [33]. Trained study staff contacted all participants randomized to the Peer TeleHCV arm (N = 100) using the participant’s preferred contact method, typically text message, phone call, or social media direct message. If unable to reach a participant, research assistants contacted peers to locate the participant through in-person outreach and connect the participant via telephone to the research assistant to complete the survey in private. A sample of Peer TeleHCV study participants also participated in qualitative interviews examining overall experience of the model, which will be reported in a forthcoming paper.

## Results

In the clinicians focus group (n = 5), the mean age was 38 (SD 4.7), 60% were male, and 100% were White. For the focus group with peers (n = 7), the mean age was 43 (SD 6.9), 71% were female, and 100% were White, with 14% also reporting that they were American Indian or Alaska Native.

### Qualitative results

Three major themes with sub-themes emerged from the interview data: key peer-level factors, key clinician-level factors, and key system and technological factors.

#### Theme 1: key peer-level factors

Overall, the role of the peers was identified as the cornerstone of the model. Clinicians, for example, emphasized the importance of the role of peers in facilitating client engagement throughout HCV treatment. Key sub-themes regarding the role of peers in the intervention included their role in 1) facilitating stabilizing wrap-around services, 2) providing support during potentially difficult lab draws (the process of collecting blood samples in a laboratory or clinical setting for hepatitis C diagnostic testing), 3) supporting and maintaining communication by enabling technology access and conducting outreach, and 4) working as intermediaries to create a bubble of trust with clinicians.

#### Subtheme 1: peers facilitate stabilizing wrap-around services

Peers support access to stabilizing wrap-around services for patients to facilitate treatment engagement. Using their knowledge base, they serve as an “*information center”* [Peer 2] and provide referrals to basic life necessities such as housing and food.

We can get people who are currently taking their medications housed for the entirety of the time that they’re taking their medications, and we provide mobile exchanges a lot. [Peer 3]

*We are mobile about 90 percent of the time going around doing outreach. After we’ve already enrolled somebody, normally, we’re trying to check up on them daily or weekly and meet them right where they’re at. Say if somebody needs help with treatment, or they need to go and find a way to get something to eat, whatever, we ask them what they need. We help them facilitate whatever that might be. [Peer 2]*

Similarly, clinicians articulated how peers shouldered the critical responsibility of building relationships with patients to understand their needs, so that connections to resources could be made and trust could be developed:

*Anything regarding their partners having infection as well or their housing situation, that’s all been handled by the peers. I might hear about it if someone has not been able to complete their treatment because of their social circumstances and whatnot. Otherwise, I have felt a little bit disengaged from the nitty-gritty of people’s lives. [Clinician 1]*

*It just speaks to how critical the peers are. How they’re really doing the bulk of the work, which is our offering of free service as a clinician. [Clinician 5]*

In the words of a peer,

*We’re showing up every single day for them saying, “How are you doing? Do you need anything?” They get to build a relationship that’s not a toxic relationship. [Peer 5]*

Subtheme 2: Peers provide support during lab visits.

Phlebotomy is a routine component of HCV diagnosis and SVR12 testing but can be difficult to perform for patients with significant scarring from current or previous injection drug use (IDU).) (e.g., multiple attempts to find a suitable vein). Peers expressed concern for patient experiencing these difficulties and as well as the stigma they often encountered in the lab setting. Peers discussed how they accompanied patients to these potentially painful or traumatizing lab draws, providing support:

*At these labs, they’re not easy. In some cases, I’ve had to go and they’ve had lower extremity draws. They’re being poked like 10 times to get enough blood for the tests, so it’s not just one or two pokes sometimes. It really is a process. I mean, we could be there an hour of this in a hard case. [Peer 3]*

*They don’t want to go to the lab and be judged. Also, they’re usually a very difficult draw. It’s not a simple lab draw for most people, and it can have trauma surrounding that in the past. Or even adding on trauma, if there’s scar tissue and the lab tech isn’t sensitive to that. [Peer 2]*

Peers shared how they built relationships with phlebotomists experienced in working with PWUD to conduct blood draws for patients involved in the study.

*I have developed relationships with the lab techs. I will ask for a certain person every time because they are the best with my clients and they will listen. If she’s not there, then I have the second person I usually ask for. [Peer 3]*

*There's a couple of people who work there who we request to come and do it because they're really good with our clients. We've made these relationships to make sure that our clients are being treated with respect. [Peer 6]*

Subtheme 3: Peers support and maintain communication by enabling technology access and conducting outreach.

A significant peer role concerned enabling communication linkages to teleHCV clinicians and maintaining contact to support medication adherence. As many participants lacked access to technology (smart phone, adequate internet, etc.) to engage in telemedicine, peers most often facilitated telemedicine appointments using their own cell phones:

*That’s the important part of the peer is when they don’t have internet—they don’t have access to a phone to have that appointment—having someone there to provide [a phone]. That has probably made the biggest difference. [Peer 7]*

Peers supported patients in accessing programs that provide cellphones, when available, to assist in maintaining communication with patients throughout their HCV treatment. Nevertheless, such resources were limited and patients’ lack of access to cellphones was seen as “their biggest barrier” *[Peer 3]* to maintaining contact.

*I cannot emphasize how often the peers have told me that [cell phone support] really made or broke their ability to keep track of the clients that are enrolled in the study. [Clinician 4]*

Peers worked diligently to maintain contact, ensuring that patients had easy access to teleHCV clinicians. A clinician noted that *“peers feel quite comfortable asking me questions, or if there’s a follow-up concern, it’s very smooth to get in contact with one another. [Clinician 5].* Respondents highlighted peers’ role in conducting outreach in the community to maintain contact and to promote medication adherence:

*I communicate with the peers to get information on how the patients are doing. If they need any additional visits or help with any of that, often I can’t get ahold of the participant, and so then I reach out to the peer. The peer is able to find them, locate them, and get them in touch with me… It largely has been the peers that have been really important in that process and in keeping the patients engaged and on-treatment. [Clinician 3]*

Subtheme 4: Peers serve as intermediaries, creating “bubbles of trust” with clinicians.

Peers mediated the relationship between patients and clinicians in intangible ways. Focus group participants noted that peers played a role in building trusting relationships between patients and clinicians:

*[Peers] create trust in a way that could not be created, initially, with a clinician; maybe over time. They sort of invite the clinician into their bubble of trust, and then warm hand-off that bubble. It probably has a greater impact on treatment initiation than even we think, I would guess. I talked to someone just a couple minutes ago doing a pre-treatment evaluation because their labs were a little bit off. They never ever go to a doctor because they feel really judged, and they said they’d go to a [Peer TeleHCV] doctor. [Clinician 2]*

The clinician added that the peer role in building trust is especially important in rural areas, where many patients had experienced stigma from past clinicians. They explained:

I think, especially if people are in an area where stigma is especially prevalent, that’s going to have an even bigger impact, in that, they probably have not had the experience of talking to a doctor who didn’t judge them about their substance use. It probably amplifies the peer *effect in that situation. [Clinician 2]*

Another clinician described how a peer advocated on behalf of the clinician with a patient who did not want to seek care because they had encountered stigma from clinicians in the past and anticipated a similar experience with the TeleHCV clinician.

The peer was able to specifically say like, “Oh I know this doctor. He’s really nice. He’s not going to bother you about anything.” So the peers have been advocating on the clinicians’ behalf to the patient, which is interesting. I didn’t really anticipate it, but I think it really helps the patients gain transfer trust. They have a lot of trust in the peer frequently, and then they can transfer that trust to the clinician, or the peers can facilitate that trust. [Clinician 5].

Peers explained that a key component of their role entailed working with patients who were distrustful of the healthcare system, advocating on their behalf and establishing reliable linkages to care:

*Our team of peer support specialists, we build relationships with all these people. They trust us when we show up because we met all these people through engagement. We know them locally in our areas for years and stuff already. When we approach them and say we’re doing a TeleHepC program and we know that they have hep C, they trust us to lead them into a good situation, go to labs, and get the treatment. [Peer 2]*

#### Theme 2: key clinician level factors

Key clinician-level factors contributing to Peer TeleHCV model implementation included (1) same-day telehealth appointments (2) dedicated time for weekly team meetings and case consults with peers, and (3) clinicians trained in working with PWUD and skilled in identifying related clinical concerns.

#### Subtheme 1: availability of same-day telehealth appointments are critical

A key clinician factor was the program’s ability to in general offer same-day telehealth visits or “walk-in” unscheduled appointments. Rapid access to a clinician, in the moment and setting in which the peer had access to the patient, was described as critical for many patients who experienced difficulties attending future appointments:

*I’ve noticed for the people who have been able to come in and have that access to a doctor right then right there has significantly changed that. A lot of the people who have tried to navigate through the community [outside of the Peer TeleHCV model], miss several appointments—were never really able to make it—so the more accessible we made it, the easier it was for people to actually get treated. [Peer 4]*

*… to get them on a call with a doctor within 20 minutes, they’re just like, “Whoa, I’ve been trying to treat my hep C for years. [Peer 1]*

*I like the fact that you can just shoot a text or send an email and let them [clinicians] know like, “Hey, this participant is here. Their labs are all done, and they’re waiting for you.” That’s just really easy and convenient. Because we can be either at a [homeless] camp or we can be in a car, or we can be at the office or in the client’s house, it doesn’t matter. We can just be there and talk to a doctor right then and there. [Peer 6]*

Subtheme 2: Weekly team meetings and case consults with peers were helpful.

Clinicians identified the weekly study team meetings as an important component for the model and an opportunity for building team cohesiveness and function between peers and clinicians. A clinician explained:

*I found it very helpful […] to have the weekly study meetings, where peers share their experiences about trying to find different participants and whatnot. I think it has helped me feel more connected to the peers, that I know what they’re doing and build some trust. I think, also, my presence, probably: I would expect from the peers, like they know who I am, and they know that they can trust me with the participants, as well. [Clinician 1]*

#### Subtheme 3: clinicians trained in working with PWUD

Several peers noted that the study clinicians’ high level of experience with PWUD facilitated engagement, created a positive interaction with the health care system, and indicated a trauma informed method of care “especially with doctors that provide or have experience with people who use drugs” [Peer 6]. Another peer expanded:

*You’re setting them up for a positive. It’s the first positive that they’re probably going to have in taking care of themselves. They actually did get to see a doctor. They actually are getting their meds. […] It just builds from there. [Peer 4]*

Peers suggested that the Peer TeleHCV model allows for patients to receive health care from a trained clinician and reduces the potential for stigmatizing treatment in an in-person office setting.

*I feel like doing it telehealth for people who are in active addiction is a really trauma informed way of doing this. There’s a lot of people that are in active addiction who won’t go to doctors because they’re treated extremely bad. They would rather not get treated for an abscess or something like that and go in to a doctor. […] They have no problem getting on a Zoom call. [Peer 2]*

Another focus group participant went on to express that this trauma-informed care enabled them to provide patients* “a sign of respect, in a sense of your input is important here, we value your experience; when you’re having trouble, we want you to be able to communicate with us.” [Clinician 4].*

#### Theme 3: key systems-level and technology elements.

Key systems-level and technology elements important for Peer TeleHCV model implementation included (1) standing lab orders, (2) challenges in working with specialty pharmacies and Medicaid managed care organizations, (3) and direct peer-to-clinician communication to streamline access to care.

#### Subtheme 1: standing lab orders facilitated access to care

Respondents remarked that standing lab orders were critical to success of the model and without them, *“there would be a lot more work for everyone, delays in care, and thinking about things like being able to get ahold of clients again [Clinician 2].* The participant opined:

*If you have them in the moment, if you can take them to the lab right away, it’s really crucial. I also would say, this [standing lab orders] is one of those things you could not do this intervention without the standing lab order with a peer-based model.” [Clinician 2]*

The respondents remarked that establishing linkages to the labs was time consuming, but worth the effort:

*I think the other barrier in setting up is around lab contracting and different labs. I think that the team spent a lot of time, each time, like making a new relationship with a new lab that’s in the community. [Clinician 2]*

Clinicians reported that a vital component of the model was choices the clinical team made in prioritizing labs to inform treatment initiation and the ability of peers to communicate these priorities when providing the standing order to lab staff. This was important in reducing the possibility of repeated lab visits for participants:

*Two things, one is lab prioritization if you have any control over that. [Peers are] having ways to communicate which labs to prioritize, so HCV RNA will always be the most important […] early on there were a lot of folks who got half their labs back, and then didn’t have enough blood for all of the specimens. But if they did like the CBC and the CMP, but they didn’t do the RNA, you still had to send them back for the RNA. Whereas this population, probably 40% or so will have a positive antibody and a negative RNA. You wouldn’t have to do any more follow-up after that if you had the RNA first, so that’s always an issue.*

Clinicians noted that dried blood spot testing could be a future improvement to the model, to reduce the need for blood draws:

*If you could do a dried blood spot test for HCV RNA after the rapid [test], or just do a reflex antibody to RNA on a dried blood spot, then again you’d have a smaller pool of targeted individuals that you’re putting those resources into engaging people with their full treatment lab evaluation. DBS has been showing studies to be preferred 4 to 1 over phlebotomy for a screening confirmation tool in other studies, and so at least the acceptability of that has been demonstrated pretty well. [Clinician 2]*

Sub theme 2: Specialty pharmacies and Medicaid managed care organizations sometimes created barriers.

Specialty pharmacies are state-licensed pharmacies focused on providing high-cost medications for high-complexity diseases. Most Oregon managed care organizations require that hepatitis C medication prescriptions are filled specialty pharmacies. Peers and clinicians alike noted challenges they encountered when working with the required specialty pharmacies that fill and mail the HCV medication prescriptions. One clinician noted that the specialty pharmacies have been “*a big barrier to getting the medications into the patients’ hands because it just involves another step….which is difficult in this population” [Clinician 3].*

*There’s the requirement for a specialty pharmacy, in general, that’s a barrier. It may well be that someone has a relationship with a pharmacy that’s local, that they would be better serviced at. Then there’s the kind of fragmentation of specialty pharmacies and the inability to use a single specialty pharmacy for all [managed care organizations] …California, for instance, has specialty pharmacy requirements, but they can use a single specialty pharmacy, so any system setting up a telehealth intervention can use the same one. [Clinician 2]*

When asked for an example of how the specialty pharmacies introduce a barrier, the clinician responded:

*I think the most common example that comes up is just that everything is ready to go. The patient is ready to start the medication, and the specialty pharmacy is trying to get ahold of the clinician. They don’t know how to, or they don’t know who to contact, or how to get ahold of peer. It just causes a delay in getting medication to the patient. [Clinician 3]*

Focus group participants’ suggestions for future implementations included greater consistency across specialty pharmacies, and the option to have the peers provide the pharmacies consent to mail medications, particularly in cases where the medications were being shipped to the peer program office. A peer related an example during a recent interaction with the specialty pharmacy:

*The representative there said—I wrote it down—the patient needs to listen to the HIPPA recording, then they have to be able to consent to ship the meds—even if they’re coming to our office. They have to, absolutely, speak with the person at least once before they will ship the meds, so I’d explained to her the nature of the population that we serve: They are—9 times out of 10—unhoused, so trying to locate them and find them, things like that, prove to be a little difficult, as was keeping their phones and stuff like that. She didn’t really want to hear anything I had to say about that. It was just cut and dry like, “No, we need to speak to this person before we will ship. [Peer 1]*

Peer focus group participants discussed challenges and inconsistent approval processes and policies when working with the Medicaid Accountable Care Organizations to obtain resources (e.g., housing funds, food, phones, etc.) for PWUD. For example, each Accountable Care Organization had differing policies about whether PWUD could access funds for temporary housing to facilitate HCV treatment retention and outcomes.

*The [*Accountable Care Organization*] in our area, they could provide the housing to people, during the time that they’re taking the medication […] I mean, they’re given funds from [Oregon Health Plan/Medicaid] to do this. At this point, I feel like every one I’ve submitted, recently, has been denied for housing, for this. Even though they have their meds on hand, they’re taking the medication, they are homeless, and they have tried other avenues to get into shelter, they’re still being denied. I feel like it’s just a blanket denial at this point. Anyone coming through me or through our program is going to get denied, regardless. [Peer 4]*

Additionally, participants shared the challenge of feeling that their role as a peer support specialist is not recognized or valued by Accountable Care Organizations:

*To me, I don’t think they [Accountable Care Organizations] recognize peers for what they do. I mean, we’re just like some person sometimes. They don’t realize how much work we really do for people and the investment we have in it. [Peer 3]*

Subtheme 3: Direct peer-to-clinician communication outside of health system platforms streamlined access to care.

Focus group participants reported that the study team had created alternate processes outside of the health system’s scheduling platform to accommodate the need for unhindered scheduling of patients:

*We have support staff assisting, but we really—to the T—outlined the language that the peers use when they call, in order to make the process seamless for the schedulers who receive the call...Each of them [schedulers] at least have had one hepatitis C client, and they know to page us, and they know to keep the client on hold. It’s certainly a trainable model, but there were significant barriers to entry for it. [Clinician 4]*

Focus group participants acknowledged that some improvements were still in process given constraints working within a system with a “*very heavy reliance upon standardization.” [Clinician 4].*

*Also, because essentially when you’re requesting models that exist outside of the system as is, oftentimes even if the model exists, there is a strong incentive for them to understand why a departure from the model is needed, why a departure from the standard is needed. And that was something that appeared a little bit more cultural in the sense of why can’t you just work on the existing templates, and why can’t you just schedule patients? … It’s a bit of unfamiliarity with the particular population that we have and the unique barriers that were present for them to have an appointment. [Clinician 4]*

Clinicians also remarked on the importance of circumventing the hospital’s existing telehealth platform by providing a direct virtual link for patients and clinicians:

*The default process is that the link or the information surrounding the appointment gets sent to [patients] through their electronic health portal. They have to access it that way, and that link is individualized. We sort of knew from the outset that would be an inherently problematic model because most of these clients were not accessing an electronic health portal… So having a universal meeting [link]—a virtual meeting platform that the peers know to utilize, that the clinicians have and can share with [peers], has been instrumental, I think, in making sure that appointments don’t get delayed and that you minimize patient barriers to entry. I think it would have been incredibly difficult had we not had this Zoom link or way to leap into an appointment adlib without a customized patient access link. [Clinician 4]*

Several respondents made suggestions for future implementations such as the need for a trained staff coordinator to manage appointment scheduling with the on-call clinician. For example,

*So that’s something that would dramatically change how difficult this is to set up, and then also the presence or absence of a specific health system side coordinator for this position who potentially could have scheduling privileges. For instance, [that] would obviate all of these scheduling challenges because you just have the point of contact be that health system’s coordinator who schedules the appointment and so on. [Clinician 2]*

Additional suggestions for future treatment model replications included potential use of a HIPPA-protected app for communicating between peers and clinicians while protecting Protect Health Information (PHI):

*About the messaging, I think that would be a really welcome addition in the future iterations of this just because it’s hard for peers to be really diligent about not texting any PHI. I think it’s good. That’s an easy time to make mistakes. As far as protecting PHI, so if we had it all in one platform, it would just take that off of the table. [Clinician 5]*

### Peer TeleHCV model peer support satisfaction survey results

Seventy-eight of 100 participants assigned to the Peer TeleHCV treatment model (78%) completed surveys 12 weeks following treatment completion. The average age was 42.2 (SD 11.5) years; most were male (59%), white (86%), and had experienced houselessness in the past 6 months (69%) (Table 1).

**Table 1:** PWUD Survey Participant Characteristics

Characteristic	Total (N = 78)	
	Mean	Standard Dev
Age	42.2	11.6
	No	%
Gender = Male	46	59.0
Race		
American Indian	4	5.1
Mixed race	2	2.6
Other	3	3.8
White	69	88.5
Hispanic ethnicity	4	5.1
Education		
< High school	24	30.8
High school/general equivalency diploma	31	39.7
Associate’s/Some college/Trade degree	22	28.2
Bachelor’s +	1	1.3
Insured	77	98.7
Unhoused in the past 12 months	54	69.2

Peer TeleHCV study participants reported high satisfaction with peer interactions in the Peer TeleHCV treatment model. Over 90% strongly agreed that they “had a say” in deciding what help they received from their peer, 92% strongly agreed that their peer told them about services to help them “stay off alcohol and/or other drugs”, and 94% strongly agreed that their peer mentor helped improve their safety related to using drugs. All agreed or strongly agreed that their peer provided “an environment where I feel safe” (Table 2).

**Table 2:** PWUD Satisfaction with Peer Support in the TeleHCV Treatment Model (n = 78)

Item	Disagree		Don’t know		Agree		No Answer	
My peer mentor was sensitive to my background	1	*1.3%*	1	*1.3%*	76	*97.4%*	0	*0.0%*

Item	Strongly disagree (%)		Disagree (%)		Agree (%)		Strongly agree (%)		No answer (%)	
My peer mentor provided an environment where I feel safe	0	*0.0*	0	*0.0*	3	*3.8*	75	*96.2*	0	*0.0*
My peer mentor spent enough time with me	0	*0.0*	0	*0.0*	3	*3.8*	75	*96.2*	0	*0.0*
I had a say in deciding what help I got from my peer mentor	1	*1.3*	2	*2.6*	4	*5.1*	71	*91.0*	0	*0.0*
My peer mentor helped me with other needs such as housing, finances, work/school	8	*10.3*	9	*11.5*	12	*15.4*	49	*62.8*	1	*1.3*
My peer mentor helped improve my safety related to using drugs	0	*0.0*	0	*0.0*	5	*6.4*	73	*93.6*	0	*0.0*

Item	Strongly disagree		Disagree		Uncertain		Agree		Strongly agree		No Answer	
My peer mentor told me about services that will help me stay off alcohol and/or drugs	0	*0.0%*	1	*1.3%*	2	*2.6%*	3	*3.8%*	72	*92.3%*	0	*0.0%*

## Discussion

In focus group interviews with peers and clinicians involved in the implementation of Oregon HOPE TeleHCV study, we found that having peers who are embedded in the community and can facilitate care and supportive services for patients, and having same-day or “walk-in” access to telemedicine clinicians who are skilled in working with PWUD, facilitates patient engagement in receiving telemedicine HCV treatment. These findings were reinforced by patient satisfaction survey responses among those receiving peer TeleHCV services, which found that patients overwhelmingly felt supported by their peers. We found that this innovative model of care continues to face system-level challenges to streamlined service delivery, including barriers related to working with Medicaid Accountable Care Organizations to obtain wrap-around supports (e.g., housing vouchers).

We found that peers were integral in the initial engagement of patients, facilitating access to telemedicine, maintaining contact for adherence and retention, and facilitating linkages to wrap-around supports. Peers guided Peer TeleHCV study participants to services for life necessities such as food and housing and navigated insurance enrollment barriers. Consistent logistic support and tangible connection to services has been documented in the literature as a strength of peer supported programs [19]. Peers provided technology and support accessing telemedicine, and maintained contact with patients by conducting outreach and staying in communication to assist patients in completing treatment. Peers and clinicians also noted how difficult and potentially traumatizing lab draws can be for PWUD. Our research compares favorably with other work that has found venipuncture to be a difficult aspect of HCV treatment for PWUD and requiring attention and care [34,35]. Peers built relationships with skilled lab technicians and provided the support needed for patients to follow through with phlebotomy.

Peers with lived experience of substance use experience can address barriers related to trust in medical system for PWUD [36,37]. Respondents in our study, especially clinicians, emphasized that peers bridged a critical gap between patients and clinicians. These findings are consistent with other research which suggests that peers foster communication between patients and clinicians and keep patients engaged in treatment [19]. In a review of the peer navigator literature, Mailloux et al. [38] found that peers assist in patient-clinician communication and engender trust in medical personnel.

Our study also demonstrates that the speed with which patients are able to connect with clinicians is of vital importance to initiating treatment and the overall success of the model. Study results can be situated in a small but growing literature on the need to adapt the current medical model for PWUD by engaging them outside of primary care offices and providing same-day care [39]. As a result of COVID 19, some important system changes have occurred for individuals seeking MOUD (medications for opioid use disorder) services. For example, a 24/7 “telebridge” clinic was established in Rhode Island where individuals seeking buprenorphine treatment were connected real-time with a prescriber and unobserved initiation [40]. In Oregon, The Harm Reduction and Bridges to Care (HRBR) Clinic transitioned from in-clinic visits to mostly same-day, online visits in the initial weeks of the COVID-19 epidemic [41,42]. This model was particularly advantageous for patients in rural communities, some of whom were driving many hours for their in-clinic appointments. Other examples include lowered barriers for MOUD including mobile units and walk-in clinics, and HCV treatment as well [6]. In a recent randomized clinical trial of PWUD with HCV higher cure rates were observed in those served by the low-threshold care as compared with referral to treatment as usual. However, efforts to extend access to care in rural areas are often handicapped by clinician shortages [43]. Syringe Service Programs also can provide accessible and non-stigmatizing care, though they are mainly located in urban areas.

Stigma is cited by PWUD as a barrier to help seeking. [44] Research indicates that healthcare professionals may demonstrate negative attitudes towards patients with substance use disorders [45] and stigma may explain the reluctance of people with OUD to initiate medical care [46]. Our findings echo these findings, as a key clinician level factor observed to be a key to access was that the study clinicians were well trained in working with PWUD and able to provide trauma-informed care. With few local clinicians in rural areas, PWUD who encounter stigma in healthcare settings may be reluctant to seek local care for HCV and value access to telemedicine clinicians for whom peers have “transferred trust.”

Our results highlighted key system level factors needed to enable rapid low-barrier entry to care. Factors included the use of standing lab orders, which enabled peers to bring patients directly to labs rather than waiting for a clinician order, and direct peer-clinician communication outside of the health system’s scheduling and telehealth platforms, which streamlined same-day access to clinicians and enabled peers to facilitate technology access to telehealth.

Other system level factors concerned challenges working with specialty pharmacies and Medicaid Accountable Care Organizations. Rigid and varied specialty pharmacy rules introduced delays in medication access. Respondents expressed frustration with inconsistent policies on how and whether people with current drug use could access wraparound resources such as temporary housing resources or phones to facilitate HCV treatment. Peers were also concerned that their role was not maximized to the patients’ advantage. The institutional and logistical obstacles they encountered have been previously reported. For example, Bonnington and Harris [47] found that peer effectiveness can be inhibited by medical organization structures. However, when peers are integrated into all facets of the HCV cascade of care, treatment outcomes improve [48].

Study findings should be interpreted in view of some limitations. Our results may not generalize to more diverse populations and health systems outside of the Oregon setting, yet the purpose of this mixed-methods study was to understand implementation of an innovative care model, rather than to be representative of more widespread populations. Additionally, we did not include public health or payer representatives in focus groups, which are likely essential partners for implementation of the Peer TeleHCV treatment model.

## Conclusions

Study findings suggest that the Peer TeleHCV treatment model offers a novel approach that transcends current healthcare barriers to treat PWUD with HCV in rural communities, where resources for both SUD and HCV treatment are limited. Our results center the role of peers as essential for the TeleHCV model’s successful implementation. Future directions and policy implications include the need to develop sustainable payment models for peers outside of SUD treatment settings. Other policy implications include addressing system changes to reduce bureaucratic barriers to same-day access to supportive care and HCV treatment. The study was successful in streamlining communication pathways between community-based peers and hospital system-based clinicians but ongoing advocacy is needed to address macro-level system changes such as utilizing a single specialty pharmacy across Accountable Care Organizations and providing equal access to member resources for PWUD.

## Data Availability

No datasets were generated or analysed during the current study.
